# Supplementary material for: FERN – a Java framework for stochastic simulation and evaluation of reaction networks
Source: BMC Bioinformatics. 2008 Aug 29;9:356. doi: 10.1186/1471-2105-9-356 (PMC2553347; doi:10.1186/1471-2105-9-356)
Supplement: Additional file 1 — FERN distribution, Version 1.3. This archive contains the FERN source code and binaries as well as documentation and example models in FernML and SBML. [file 1471-2105-9-356-S1.zip › fern/doc/javadoc/fern/tools/NumberTools.html]

NumberTools


---


|  |  |  |
| --- | --- | --- |
| **Overview** \| **Package** \| **Class** \| **Use** \| **Tree** \| **Deprecated** \| **Index** \| **Help** | |  |
| **PREV CLASS**   **NEXT CLASS** | **FRAMES**    **NO FRAMES**     **All Classes** |  |
| SUMMARY: NESTED \| FIELD \| CONSTR \| METHOD | DETAIL: FIELD \| CONSTR \| METHOD |  |


---


## fern.tools Class NumberTools

```
java.lang.Object
  fern.tools.NumberTools
```

---

``` public class NumberTools extends Object ```

Contains some common methods to handle number related data structures.

**Author:**
:   Florian Erhard

---

**Constructor Summary**

`NumberTools()`


| **Method Summary** | |
| --- | --- |
| `static int` | `argMax(Map<Integer,Integer> l)`             Gets the argmax of the given map. |
| `static double` | `avg(double[] a)`             Gets the avg of the array |
| `static double` | `calculateHistogramDistance(Map<Integer,Integer> h1, Map<Integer,Integer> h2)`             Calculates the histogram distance of two histograms. |
| `static double[]` | `convertIntToDouble(int[] a)`             Performs a deep cast of an int[]. |
| `static double[][]` | `createHistogram(Collection<double[]> data, int numBins, int... indices)`             Creates a histogram from the given data. |
| `static int[]` | `createHistogram(int[] a)`             Create a histogram for the given array. |
| `static Map<Integer,Integer>` | `createHistogramAsMap(int[] a)`             Create a histogram map for the given array. |
| `static int[]` | `createInverse(int[] a)`             Creates the inverse array to the given array. |
| `static Map<Integer,Integer>` | `createInverseAsMap(int[] a)`             Creates the inverse map to the given array. |
| `static void` | `cumSum(int[] l)`             Replaces the values in the given array with the cumulative sum of the array. |
| `static int` | `faculty(int i)`             Gets the faculty |
| `static int[]` | `getContentAsArray(BitVector bv)`             Gets the indices of the set bits of the given `BitVector` as array. |
| `static BitVector` | `getContentAsBitVector(int[] a)`             Gets the content of the given array as `BitVector`, which means, that the `BitVector` will have a size equal to the maximal value in the array and contains a 1 for each element which is contained at least once in the array |
| `static String` | `getHistogramAsString(Map<Integer,Integer> h)`             Gets a string representation of the histogram map. |
| `static int[]` | `getNumbersTo(int n)`             Gets the numbers from 0 to n in an array. |
| `static double[]` | `interpolateLinear(double interval, double[] time, double[] value)`             Performs a linear interpolation of the values. |
| `static double` | `interpolateLinear(double desiredTime, double beforeTime, double afterTime, double beforeValue, double afterValue)`             Interpolates a value |
| `static String[]` | `inverse(String[] a)`             Inverts the given array in place and returns it. |
| `static String` | `join(String[] a, String glue)`             Joins the array with the given glue to one String. |
| `static Map<Integer,Integer>` | `loadHistogram(File file)`             Reads a previously saved histogram from a file. |
| `static int` | `max(Iterable<Integer> l)`             Gets the maximal value within the given `Iterable`. |
| `static int` | `min(Iterable<Integer> l)`             Gets the minimal value within the given `Iterable`. |
| `static void` | `saveHistogram(Map<Integer,Integer> histo, File file)`             Writes a histogram to a file. |
| `static void` | `shuffle(int[] a)`             Shuffles the content of the given array. |
| `static double` | `stddev(double[] a)`             Gets the standard deviation of the array |
| `static int` | `sum(int[] l)`             Gets the sum of the values in the array. |
| `static double[]` | `toArray(Collection<Double> list)`             Copies the values of the given collection and unboxes them to an array. |
| `static int[]` | `toArray(Collection<Integer> list)`             Copies the values of the given collection and unboxes them to an array. |

**Methods inherited from class java.lang.Object**

`clone, equals, finalize, getClass, hashCode, notify, notifyAll, toString, wait, wait, wait`

**Constructor Detail**

### NumberTools

```
public NumberTools()
```


**Method Detail**

### faculty

```
public static int faculty(int i)
```

:   Gets the faculty

    :   **Parameters:**: `i` - the number **Returns:**: faculty(i)

---


### max

```
public static int max(Iterable<Integer> l)
```

:   Gets the maximal value within the given `Iterable`.

    :   **Parameters:**: `l` - the iterable **Returns:**: the maximal number

---


### min

```
public static int min(Iterable<Integer> l)
```

:   Gets the minimal value within the given `Iterable`.

    :   **Parameters:**: `l` - the iterable **Returns:**: the minimal number

---


### argMax

```
public static int argMax(Map<Integer,Integer> l)
```

:   Gets the argmax of the given map. The argmax is the key whose
    value is the maximum within the values.

    :   **Parameters:**: `l` - map **Returns:**: argmax(map)

---


### sum

```
public static int sum(int[] l)
```

:   Gets the sum of the values in the array.

    :   **Parameters:**: `l` - the array **Returns:**: sum of the values

---


### cumSum

```
public static void cumSum(int[] l)
```

:   Replaces the values in the given array with the cumulative sum
    of the array.

    :   **Parameters:**: `l` - the array

---


### toArray

```
public static int[] toArray(Collection<Integer> list)
```

:   Copies the values of the given collection and unboxes them to an array.

    :   **Parameters:**: `list` - collection **Returns:**: array with the same values

---


### toArray

```
public static double[] toArray(Collection<Double> list)
```

:   Copies the values of the given collection and unboxes them to an array.

    :   **Parameters:**: `list` - collection **Returns:**: array with the same values

---


### createInverse

```
public static int[] createInverse(int[] a)
```

:   Creates the inverse array to the given array. In the inverse array a' of a
    following condition holds:

    `a[i]=j <=> a'[j]=i

    Be careful with sparse arrays, the inverse array could be very large (prefer
    createInverseAsMap(int[]). It should preferably be used
    with bijective arrays.

    :   Parameters:: a - the array Returns:: the inverse`

---


### createInverseAsMap

```
public static Map<Integer,Integer> createInverseAsMap(int[] a)
```

:   Creates the inverse map to the given array. In the inverse map a' of a
    following condition holds:

    `a[i]=j <=> a'.get(j)=i

    :   Parameters:: a - the array Returns:: the inverse map`

---


### createHistogram

```
public static double[][] createHistogram(Collection<double[]> data,
                                         int numBins,
                                         int... indices)
```

:   Creates a histogram from the given data. The returned array matrix contains in the
    first column the x axis (if the first index of the array matrix denotes the column index)
    The other columns contain the frequencies of values containing to the corresponding
    bin of the x axis.

    :   **Parameters:**: `data` - data to create the histogram for: `numBins` - number of bins, the return array matrix will have numBins rows: `indices` - the indices of the data to create the histogram for **Returns:**: histogram

---


### createHistogram

```
public static int[] createHistogram(int[] a)
```

:   Create a histogram for the given array. Be careful with sparse arrays, prefer
    `createHistogramAsMap(int[])`.

    :   **Parameters:**: `a` - the array to create a histogram for **Returns:**: histogram for the array

---


### createHistogramAsMap

```
public static Map<Integer,Integer> createHistogramAsMap(int[] a)
```

:   Create a histogram map for the given array.

    :   **Parameters:**: `a` - the array to create a histogram for **Returns:**: histogram for the array

---


### saveHistogram

```
public static void saveHistogram(Map<Integer,Integer> histo,
                                 File file)
                          throws IOException
```

:   Writes a histogram to a file. Then it can be loaded by  `loadHistogram`.

    :   **Parameters:**: `histo` - the histogram to save: `file` - the file **Throws:**: `IOException`

---


### loadHistogram

```
public static Map<Integer,Integer> loadHistogram(File file)
                                          throws IOException
```

:   Reads a previously saved histogram from a file.

    :   **Parameters:**: `file` - the file **Returns:**: the histogram **Throws:**: `IOException`

---


### getHistogramAsString

```
public static String getHistogramAsString(Map<Integer,Integer> h)
```

:   Gets a string representation of the histogram map.

    :   **Parameters:**: `h` - histogram **Returns:**: string representation

---


### getContentAsBitVector

```
public static BitVector getContentAsBitVector(int[] a)
```

:   Gets the content of the given array as `BitVector`, which means, that
    the `BitVector` will have a size equal to the maximal value in the
    array and contains a 1 for each element which is contained at least once in the array

    :   **Parameters:**: `a` - array **Returns:**: `BitVector> for the array`

---


### getContentAsArray

```
public static int[] getContentAsArray(BitVector bv)
```

:   Gets the indices of the set bits of the given `BitVector` as array.

    :   **Parameters:**: `bv` - the `BitVector` **Returns:**: array with the set indices

---


### shuffle

```
public static void shuffle(int[] a)
```

:   Shuffles the content of the given array.

    :   **Parameters:**: `a` - array

---


### convertIntToDouble

```
public static double[] convertIntToDouble(int[] a)
```

:   Performs a deep cast of an int[].

    :   **Parameters:**: `a` - the array **Returns:**: the casted array

---


### getNumbersTo

```
public static int[] getNumbersTo(int n)
```

:   Gets the numbers from 0 to n in an array.

    :   **Parameters:**: `n` - maximal number **Returns:**: array of numbers

---


### calculateHistogramDistance

```
public static double calculateHistogramDistance(Map<Integer,Integer> h1,
                                                Map<Integer,Integer> h2)
```

:   Calculates the histogram distance of two histograms.

    For reference see Cao & Petzold, Accuracy limitations and the measurement of errors in
    the stochastic simulation of chemically reacting systems, Journal of Computational Physics 212 (2006) 6�24

    :   **Parameters:**: `h1` - first histogram: `h2` - second histogram **Returns:**: histogram distance

---


### avg

```
public static double avg(double[] a)
```

:   Gets the avg of the array

    :   **Parameters:**: `a` - array **Returns:**: avg(array)

---


### stddev

```
public static double stddev(double[] a)
```

:   Gets the standard deviation of the array

    :   **Parameters:**: `a` - array **Returns:**: stddev(array)

---


### inverse

```
public static String[] inverse(String[] a)
```

:   Inverts the given array in place and returns it.

    :   **Parameters:**: `a` - array **Returns:**: inverted array

---


### join

```
public static String join(String[] a,
                          String glue)
```

:   Joins the array with the given glue to one String.

    :   **Parameters:**: `a` - array: `glue` - glue **Returns:**: joined array

---


### interpolateLinear

```
public static double[] interpolateLinear(double interval,
                                         double[] time,
                                         double[] value)
```

:   Performs a linear interpolation of the values. The array time and the array
    values must have the same length which means that each element in time is associated
    with one element in values. The time interval is rescaled to match the given
    interval and the corresponding values are interpolated.

    :   **Parameters:**: `interval` - the interval: `time` - time array: `value` - value array **Returns:**: interpolated value array

---


### interpolateLinear

```
public static double interpolateLinear(double desiredTime,
                                       double beforeTime,
                                       double afterTime,
                                       double beforeValue,
                                       double afterValue)
```

:   Interpolates a value

    :   **Parameters:**: `desiredTime` -: `beforeTime` -: `afterTime` -: `beforeValue` -: `afterValue` - **Returns:**: interpolated value


---


|  |  |  |
| --- | --- | --- |
| **Overview** \| **Package** \| **Class** \| **Use** \| **Tree** \| **Deprecated** \| **Index** \| **Help** | |  |
| **PREV CLASS**   **NEXT CLASS** | **FRAMES**    **NO FRAMES**     **All Classes** |  |
| SUMMARY: NESTED \| FIELD \| CONSTR \| METHOD | DETAIL: FIELD \| CONSTR \| METHOD |  |


---
